# Size-Dependent Affinity of Glycine and Its Short Oligomers to Pyrite Surface: A Model for Prebiotic Accumulation of Amino Acid Oligomers on a Mineral Surface

**DOI:** 10.3390/ijms19020365

**Published:** 2018-01-25

**Authors:** Rehana Afrin, Narangerel Ganbaatar, Masashi Aono, H. James Cleaves, Taka-aki Yano, Masahiko Hara

**Affiliations:** 1 Chemical Evolution Lab Unit, Earth-Life Science Institute (ELSI), Tokyo Institute of Technology, 2-12-1-IE-1 Ookayama, Meguro-ku, Tokyo 152-8550, Japan; namynamy02@gmail.com (N.G.); aono@sfc.keio.ac.jp (M.A.); hcleaves@elsi.jp (H.J.C.); yano@echem.titech.ac.jp (T.Y.); masahara@echem.titech.ac.jp (M.H.); 2 School of Materials and Chemical Technology, Tokyo Institute of Technology, 4259 Nagatsuta, Midori-ku, Yokohama 226-8502, Japan; 3 Faculty of Environment and Information Studies, Keio University, 5322 Endo, Fujisawa-shi, Kanagawa 252-0882, Japan

**Keywords:** pyrite, mineral surface, atomic force microscopy, unbinding force measurements, glycine, oligo-glycines, peptides, single molecule interaction, origins of life

## Abstract

The interaction strength of progressively longer oligomers of glycine, (Gly), di-Gly, tri-Gly, and penta-Gly, with a natural pyrite surface was directly measured using the force mode of an atomic force microscope (AFM). In recent years, selective activation of abiotically formed amino acids on mineral surfaces, especially that of pyrite, has been proposed as an important step in many origins of life scenarios. To investigate such notions, we used AFM-based force measurements to probe possible non-covalent interactions between pyrite and amino acids, starting from the simplest amino acid, Gly. Although Gly itself interacted with the pyrite surface only weakly, progressively larger unbinding forces and binding frequencies were obtained using oligomers from di-Gly to penta-Gly. In addition to an expected increase of the configurational entropy and size-dependent van der Waals force, the increasing number of polar peptide bonds, among others, may be responsible for this observation. The effect of chain length was also investigated by performing similar experiments using l-lysine vs. poly-l-lysine (PLL), and l-glutamic acid vs. poly-l-glutamic acid. The results suggest that longer oligomers/polymers of amino acids can be preferentially adsorbed on pyrite surfaces.

## 1. Introduction

All life on Earth derives from a small cohort of cellular ancestors that emerged some ~3.5 billion years ago [1,2,3]. It is generally held that the raw materials, e.g., amino acids, nucleotides, or lipids, for the formation of primitive cells must have accumulated from abiotic synthesis. Many of the most important molecules in biochemistry are polymeric, for example, nucleic acids and proteins. The efficient polymerization of compounds, such as amino acids from a complex prebiotic organic mixture, may have required some sort of molecular selection process. 

Selective mechanisms for the accumulation of amino acids or nucleotides remain crucial issues for chemical evolution prior to the emergence of proto-cells. Many origins of life models assume that the initial chemical organization occurred in small bodies of surface water, which were periodically dried and refilled [4,5]. When this evaporation and refilling process was repeated, the concentration of organics became high enough for polymerization to occur [4,6,7]. This mechanism of non-selective accumulation could have increased the total concentration of abiotically synthesized molecules, but the encounter efficiency of specific kinds of compounds, e.g., amino acids, would still be vanishingly low, as other types of compounds would be similarly concentrated. 

The adsorption of small molecules to specific kinds of mineral surfaces for selective concentration, catalytic polymerization, protection from UV light, etc. [2] is one potential solution to this problem. The adsorption of molecules from a dilute solution to mineral surfaces would have greatly increased their effective localized concentrations. If the mineral is fine-grained or highly porous, then the total effective area for adsorption can be very large. The notion of mineral adsorption as a means of concentrating and protecting prebiological organic compounds can be attributed to Bernal [8,9,10,11,12], though it was later elaborated by Cairns-Smith who suggested the accurate self-replicating property of minerals as a possible carrier of genetic information [13]. Some mineral surfaces, such as calcite [14], have locally charged sites at the atomic scale and have the capacity to adsorb small molecules with precise and regular orientation according to the complementarity of electrostatic charge distributions and molecular shape. Both types of complementarity are prerequisite conditions for effective binding and catalysis. 

Various methods, for example, zeta-potential measurements [15], X-ray Photoelectron Spectroscopy (XPS) [16], and catalytic degradation of peptides [17], have been used to study the adsorption of amino acids to mineral surfaces. Atomic force microscopy (AFM) is a direct method allowing for the measurement of the unbinding force (related to the amount of energy required to remove an adsorbed molecule from a surface) of a single amino acid molecule from a local site on a mineral surface. AFM is especially suited for recording the frequency of unbinding events and the magnitude of the required force to separate non-covalently bonded molecular pairs. A general discussion of such force measurements using AFM can be found in [18,19]. 

Pyrite has received considerable attention as a natural mineral that may have assisted molecular organization during chemical evolution. Pyrite is a composite of iron and sulfur (FeS_2_), which, due to its extremely low solubility product (*K*_sp_) value, forms readily where reduced sulfur and iron are present in the environment. Pyrite is a common mineral in many environmental settings, including on-axis hydrothermal vents. 

Wächtershäuser et al. proposed that pyrite-hosting hydrothermal environments with natural pH and temperature gradients could have been ideal sites for the origin of metabolic systems, which eventually complexified to give rise to life [20,21]. The presence of iron-sulfur cores in several modern proteins, such as ferredoxins and rubredoxins, has been offered as additional evidence for the “iron-sulfur” world hypothesis [22]. Pyrite has a simple cubic crystal form and its three orthogonal surfaces have exactly the same arrangement of atoms. The surface of natural pyrite has various defect sites where either iron or sulfur ions are exposed and can serve as adsorption sites for various ionic species. The adsorption of amino acids on the surface of pyrite has been studied experimentally and theoretically [12,23,24,25,26,27,28]. 

Stimulated by such speculations, experimental and theoretical investigations on the adsorption mechanism of amino acids onto pyrite surface have been carried out [15,29,30,31]. Bebie et al. measured the zeta-potential, which is proportional to the electrophoretic mobility of pyrite powder in the presence and absence of several amino acids, in particular, Gly, and interpreted the change in zeta-potential as being due to Gly binding though at a lower level [15]. We have also published recent results on the interaction of pyrite surface with lysine tethered to an AFM probe through covalent cross linkers [31]. 

Studies on the interaction of amino acids with mineral surfaces are also of significant interest. For example, recent work by Razvag et al. examined the adsorption of lysine and several other amino acids to a crystalline silicon wafer or mica [32]. They used an AFM in the force mode and reported the most probable single molecule unbinding force in an aqueous buffered solution to be 68 pN for leucine, 69 pN for lysine, and 219 pN for phenylalanine, all in the typical force range of non-covalent bond disruption. Lambert et al. and Meng et al. [33] studied the adsorption of Gly on silica. Lambert et al. [27] and Marshall-Bowman et al. [17] also studied the effect of pyrite on the hydrolysis of polypeptides. The role of mineral-organic interactions in the origins of life has been reviewed by Cleaves et al. [34]. 

The interaction of glutamate with the surface of TiO_2_ was studied by Jonsson et al. using potentiometric titration and batch adsorption experiments over a wide range of pH values, ligand-to-solid ratios, and ionic strengths [35]. Hazen et al. reported the selective interaction of l- and d-amino acids with calcite surfaces [1]. De la Cruz-López et al. [36] using scanning electron microscopy, infrared spectroscopy, and semi-empirical molecular computational simulations, found that Gly self-assembled on pyrite with and without exposure to UV radiation. 

Since the adsorption of amino acids to pyrite surfaces is often mediated by non-covalent interactions, adsorbed amino acids may have the freedom to move around on the mineral surface. Translational movement of bound molecules on the surface could facilitate the encounter of monomers for oligomerization. Ideally then, the unbinding force should not be too large to prevent easy movement of the molecules on the surface, and yet strong enough to keep them near the surface. Weak binding with the retained mobility of adsorbates would be entropically favorable for adsorption. 

We herein measured the range of unbinding forces of amino acids adsorbed on pyrite surfaces to obtain an estimate of the strength of non-covalent surface interactions that may lead to peptide formation. AFM is especially well-suited to making these types of measurements at the single molecule level. 

We used AFM to measure the magnitude of mechanical force needed to disrupt the adhesive bonds once they are formed and the relative frequency of formation of such bonds between a pyrite surface and molecules that were tethered to the AFM probe through long chain cross-linkers. Using an AFM, it is possible to study adsorption in liquid. When compared with other methods commonly used in binding studies, the AFM method has the advantage of being able to probe early binding events that are completed within tens of milliseconds after the first encounter between the adsorbant and adsorbate. This eliminates the complication of measuring the contribution of possible long term secondary effects, such as aggregation in bulk experiments or chemical bond formation, which might occur after initial adsorption. All of the experiments in this work were performed at pH 7.4 and at 25 °C. 

## 2. Results

### 2.1. Imaging of Pyrite

**Figure 1:**
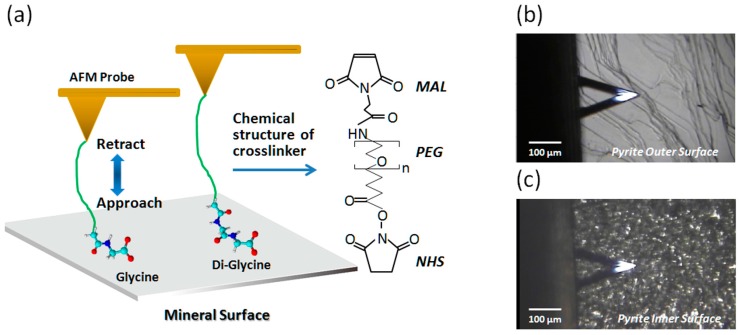
Schematic representation of this work. (**a**) The concept of stabilized binding of longer oligomers on a mineral surface eventually increasing the surface concentration of polymerized molecules. Amino acids and short oligopeptides were tethered to a hetero bi-functional polyethylene glycol (PEG) crosslinker through aminolysis of the NHS part and as a consequence, Gly and the Gly oligomers used in this study have one negative charge but no positive charge, while Lys and PLL contain additional positive charges. Optical microscopic images of the pyrite surface during force curve measurements on the atomic force microscopy (AFM); (**b**) outer surface of pyrite with steps and terraces clearly visible; and, (**c**) inner surface of pyrite showing different features than that of outer surface.

Figure 1a depicts the force measuring mode of AFM on Gly and di-Gly, together with the chemical formula of the crosslinker, Poly(ethylene glycol) (*N*-hydroxysuccinimide 5-pentanoate) ether *N*′-(3-maleimidopropionyl)aminoethane (MAL-PEG-NHS, PEG crosslinker). In Figure 1b,c, optical images of the outer and newly exposed inner surface of a cut pyrite plate (see Methods and Materials) are given with the shadow of AFM cantilever. The optical image in Figure 1b shows an abundance of terraces, step-edges, and kink-like regions on the outer surface, whereas in Figure 1c, the reflection of the illumination light accentuates the rugged inner surface.

**Figure 2:**
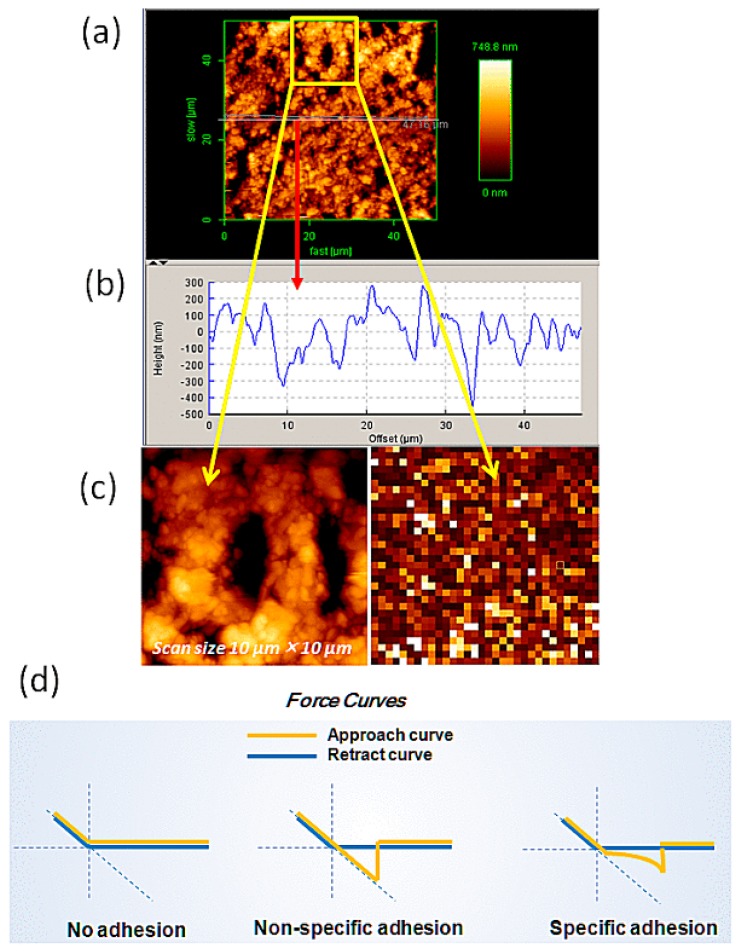
AFM topography of a cut pyrite inner surface taken using the contact mode. (**a**) Height image of 50 µm × 50 µm area, the vertical color scale is from 0 to 748.8 nm; (**b**) cross section of the surface area of pyrite along the center horizontal line in (**a**) under AFM. The surface was not quite as flat as a cleaved mica or graphite surface but it was possible to perform force curve measurements using an AFM probe; (**c**) a zoom-in of the height image and its force map image from (**a**) (marked as yellow box); and, (**d**) schematic examples of force curves for no-adhesion, non-specific adhesion and specific adhesion respectively.

The topographic image of the inner and outer most surface of a pyrite thin plate was taken by AFM under air and in solution using the contact mode with hard and soft cantilevers, respectively. In both cases, the surface appeared similar. The inner surface of the pyrite was found to be rather rough (Figure 2a) as expected from the image in Figure 1c. The average cross sectional height was in the range of 500–800 nm (Figure 2b). The natural outer surface had a roughness of about ±10–20 nm since it was originally a crystal face. An advantage of AFM is the ability to directly measure interactions at localized regions presenting specific geometrical characteristics, such as these surface imperfections. In Figure 2d, we present three typical force curves observed in AFM force mode experiments, such as this work. We tried to obtain curves that represent specific unbinding events as distinguished from no adhesion or non-specific adhesion with zero extension of the PEG spacer. 

The relative size comparison between the height and the angle of the probe and that of a prominent crevice or dent on the pyrite surface was considered. Since the height of the tip (2.9 µm) and the opening angle (72°), respectively, are longer and narrower than those of the dent, force measurements were usually performed with little difficulty. Another problem in force curve measurements is the appearance of weak or strong adhesion peaks immediately after the release of the tip from the pyrite surface. These adhesive events are due to the limited local nature of the pyrite surface, because in some cases, 500–1000 force curves were obtained free of initial adhesion peaks, but in some other cases, these adhesive peaks appeared more frequently. We avoided the inclusion of these peaks in unbinding events for analysis by restricting the collection of peaks that appeared between 15 and 60 nm of sample extension range using a custom-written user procedure for AR 14.23.153 (Asylum) on IGOR Pro (WaveMetrics Inc., Lake Oswego, OR, USA). The maximally extended length of the PEG spacer of MW ~4000 would be ~30–40 nm. Allowing for a possible heterogeneity in the MW of the PEG crosslinker, we collected force peaks up to 60 nm extension. 

### 2.2. Force Measurements with Gly and Its Oligomers in 1/10× PBS

**Figure 3:**
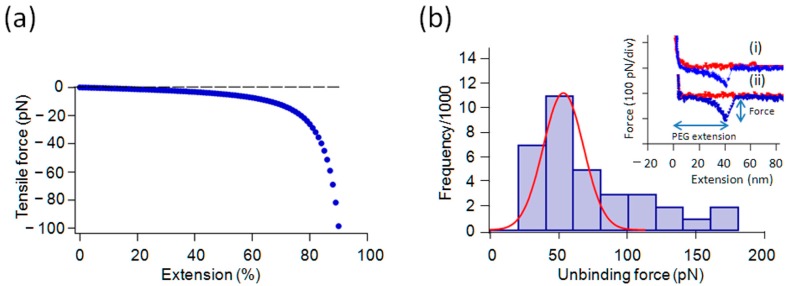
Theoretical polymer extension curve and results of Gly and di-Gly unbinding. (**a**) An expected force-extension curve of flexible polymer modeling the PEG spacer behavior up to 95% extension of the total contour length (based on the worm-like chain model [38], with the persistence length of 0.1 nm); (**b**) (inset curves) force curves of monomeric Gly (i) and di-Gly (ii) in the approach regime to the pyrite surface (red) and the retracting regime from the surface (blue). The ordinate is the force detected as the product of the cantilever deflection (nm) and its spring constant (N/m). The initial non-linear deflection due to the extension of the PEG crosslinker is abruptly terminated and the force jumped to zero. The final rupture force was identified as the unbinding force. Only those force peaks observed between 15 and 60 nm of extension were considered to be representing positive interactions and collected for further analysis; (main figure) Histogram of unbinding force for di-Gly, which gave the most probable force (MPF) of ~50 pN as the mode of Gaussian fitting curve in red. The frequency of observing unbinding force peaks matching our criteria was too low for monomeric Gly to construct a meaningful histogram.

Force spectroscopy was performed against the outer and inner surface of the cleaned pyrite plate under 1/10× phosphate buffered saline (PBS) (original PBS contained 12 mM phosphate and 140 mM NaCl/KCl) using tips modified with samples in addition to unmodified tips where only PEG crosslinkers were attached. A modified cantilever was brought to the pyrite surface and was retracted after touching the surface. Figure 3a gives the force-extension curve of the worm-like chain (see the legend to Figure 3) as a model for PEG spacer extension. The force curves shown in Figure 3b (i) and (ii) are for Gly and di-Gly taken with modified tips with a small set-point voltage. These show non-linear extensions that are mainly due to the PEG crosslinker (abscissa) up to ~30–40 nm before a sudden breakdown of the curve from ~60–100 pN to zero force level. The maximum force before the breakdown was interpreted as the unbinding force. In a typical experiment, one approach (red) and retraction (blue) cycle took a few seconds over the distance of 0.5–1 µm. Assuming the height of the PEG cross linker brush on the cantilever is in the range of 10–20 nm, as estimated from its radius of gyration in free solution [37], the allowed time for binding was ~20 ms. During the extraction process, any unbinding that occurred while the tensile force of the PEG spacer was less than ~10 pN would not be detected as an unbinding event. Therefore, sample binding must occur within 40–60 ms to be detected. As a reference, a freshly cleaved mica substrate was used in the same environment to verify the cleanliness of the cantilever. 

We noticed a distinct skewness in the histogram in Figure 3 and others in this paper as has been often observed in other work (e.g., ref. [32]). The origin of the skewness has been actively debated, but is not entirely clear [39]. We adopted a common procedure of fitting the distribution around the highest peak in the histogram by a Gaussian curve and obtained its mode as the most probable force in a given histogram. In a Gaussian curve, the mode, mean and median coincide.

### 2.3. Effect of Salt Concentrations on the Interaction

The force measurement experiments were also performed in various concentrations of salt solution prepared by diluting the buffer solution (from 1× to 1/10× PBS diluted with Milli-Q water) at room temperature. Only a few unbinding events were detected with a clear force peak in the retraction regime of the force curve in an original PBS at pH 7.4 and room temperature. However, the frequency of unbinding events with a clear indication of single event rupture for Gly increased slightly and more notably for its oligomers in 1/10× diluted PBS and Milli-Q water, respectively. We interpreted this as evidence that the interaction between Gly and Gly-oligomers with the surface is at least partially ionic and performed further experiments in a dilute PBS. The mean unbinding force in 1/10× PBS for Gly was between 40–60 and 50 pN for di-Gly (see the histogram for di-Gly in Figure 3B). This difference was not considered significant. The frequency of observation of positive interactions for Gly was low and a histogram was not constructed. Using an electrophoretic method, Bebie and Schoonen showed that Gly interacted with pyrite powder only weakly [15]. We have also previously observed a low frequency of interaction between Gly and pyrite [31]. 

The initial extension part of the actual force curves was not exactly like the worm-like chain (or an entropic chain) (WLC) model [38] extension for unknown reasons (Figure 3a). Experimental curves often had a higher force in the middle region when compared with the theoretical curve. This may be due to some force dissipating process involved in the pulling out of PEG from the crowded entanglement conditions on the tip, or the presence of very weak interactions between PEG and substrate are significant. These hypotheses are left for future studies.

### 2.4. Tri-Gly and Penta-Gly

**Figure 4:**
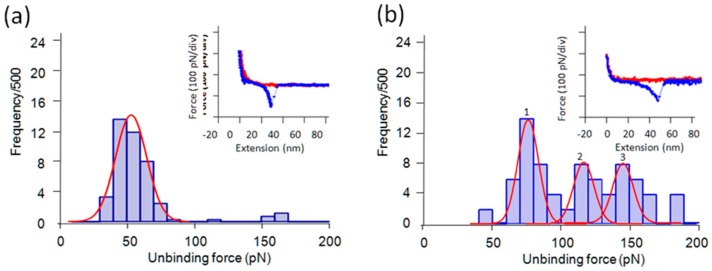
Force curves and histograms of two oligo-Glys (**a**) Tri-Gly (**b**) Penta-Gly. Three peaks were identified in the penta-Gly histogram with the mean of 70 pN (peak 1), 110 pN (2), and 140 pN (3). The higher force peaks were considered to correspond to cooperative unbinding of two or more binding units.

Using modified tips with tri-Gly or penta-Gly, we obtained more frequent binding events on the pyrite surfaces (Figure 4). The shapes of the curves were similar in both cases and not much different from those given in Figure 3, but significant differences were observed in the frequency of binding interactions and MPF obtained as the mean of the Gaussian fitting curves. 

From the histogram based on the force curves, such as that shown in Figure 4, the MPF was determined to be ~55 and ~76 pN, respectively, for tri-Gly and penta-Gly. Since all the experiments were performed under the same conditions, the MPF for different molecular species can be regarded as having been obtained under similar loading rates. The difference in the observed MPF (mode) in 95% confidence level [40] were: 21 ± 5 pN for the first peak of penta-Gly (*n* = 36, σ (standard deviation) ~10 pN) vs. tri-Gly (*n* = 38, σ = 11 pN), and 26 ± 7 pN for penta-Gly against di-Gly (*n* = 32, σ =18 pN). The difference between tri-Gly and di-Gly was marginal being 5 ± 5 pN at 80% confidence level.

The MPFs for di-, tri-, and penta-Gly tend to increase along with the oligomer length. The frequency of observing an unbinding force was lower for di-Gly and higher for tri-Gly and penta-Gly. Especially in the case of penta-Gly, there were two extra peaks in the higher force regions of the histogram. The interpretation of this result is that the number of binding sites increases according to the number of Gly residues. In addition to the size dependent van der Waals force, certain residue number-dependent factor(s), most likely specific functional groups of a peptide group, seem to enhance the frequency of adsorption and possibly the unbinding forces (see Discussion). 

**Figure 5:**
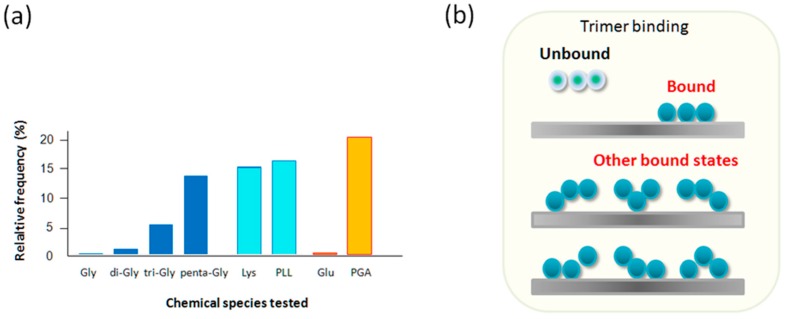
Relative frequency of binding events. (**a**) The relative frequencies of observing force peaks satisfying our criteria were, less than 1%, 3–5%, 7–10%, 10–15% for Gly, di-Gly, tri-Gly, and penta-Gly, respectively. The data on Lys, PLL, Glu, and PGA are also shown indicating enhanced binding of positively charged molecular species for Lys and length dependency for the rest; and, (**b**) schematic explanation on the increased statistical factor for binding of tri-Gly, which is assumed to have three binding sites (including the N-terminal amide) with corresponding number of acceptor sites on pyrite. There are considered to be seven possible bound states with different configurations against on unbound state.

In Figure 5, the relative frequency of observing unbinding events for Gly oligomers are given together with those for Lys, PLL, Glu, and PGA. One reason to explain the observed frequency increase from Gly to penta-Gly is in the statistical factor, *W*, for a ligand having multiple binding sites. According to this factor, a ligand with three identical and independent binding sites (*n* = 3), for example, has one unbound vs. seven bound states with different configurations. If *n* = 5, the number increases to 31 (=2^5^ − 1). This statistical factor is directly related to the configurational entropy change on binding as Δ*S* = *k*_B_ ln*W,* where *k*_B_ and *W,* are, respectively, the Boltzmann constant and the number of possible configurations. An increase in entropy has a certain contribution to the stabilization of bound states, together with energetic stabilization of bound species.

As for the increase in unbinding force, if unbinding under a tensile force occurs sequentially along the chain, the unbinding force would show only a relatively small length dependency. Therefore, for penta-Gly, a certain degree of interdependence between binding sites must be at play (see Discussion).

It should be noted that there is an upper limit to the relative frequency of observing interaction due to experimental setups that are required for measuring single molecule interactions, such as the finite probability of interaction within the contact time between the probe and the substrate, and the loss of information obscured by the initial adhesion peaks. 

### 2.5. Lys and PLL, Glu vs. PGA

**Figure 6:**
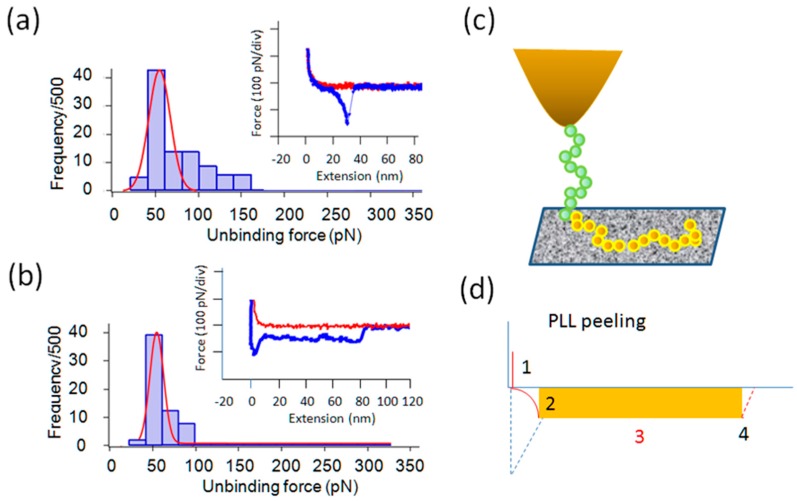
Results of lysine (Lys) and poly-l-lysine (PLL). (**a**) Histogram and force curve for Lys interaction with pyrite; (**b**) Histogram and polymer peeling/pull-out type force curve with a plateau for PLL. The binding frequency was relatively high for both Lys and PLL; (**c**) schematic explanation of binding and unbinding (peeling in this case) of linearly adsorbed PLL (green for PEG and brown for PLL); and, (**d**) Schematic of extension of free polymer chain. From 1 to 2 (blue dotted line: non-specific interaction), peeling of polymer chain from 2 through 3 to the final detachment of the chain from the substrate at 4. The integrated area (orange box) gives the total work of chain peeling.

To test the length dependence factors that could influence either the binding frequency and/or unbinding force, we performed similar force curve measurements on poly-l-Lysine (PLL) vs. Lys. The results are shown in Figure 6. The length dependency in this case was studied using a PLL of MW ~70,000–150,000. Both Lys and PLL gave relatively high binding frequencies, indicating positive charges and the chain length both promote binding of these molecules, even though they possess a negative charge. Most of the PLL force curves had an extended plateau, which is often attributed to the peeling process of adsorbed chain molecules to a solid surface or a pull-out event of a chain from polymer entanglements (Figure 6b inset) [41]. In this case, because the extension length was appreciably longer than the PEG spacer, we concluded that a part of the plateau was due to PLL peeling/pull-out. We performed a similar experiment using Glu and PGA. For Glu, the interaction with pyrite was rare, but for PGA, we obtained a high level of interaction as summarized in Table 1 and in Figure 5. It is not surprising that monomeric Glu, having two negative charges, showed little binding. It was, however, rather surprising that PGA, having opposite charges to PLL, bound to the pyrite surface. We tentatively interpret this result as indicating that the oxidized pyrite surface has both positively charged and negatively charged patches. Small monomeric Gly and Glu probably fail to find appropriate binding spots. Since PLL and PGA have short stretches of nonpolar methylene bridge groups, the possibility of a partly hydrophobic interaction with pyrite surface should also be considered in the future studies. There are chain length dependent binding events for Gly oligomers, Lys vs. PLL and Glu vs. PGA. Thus longer oligomers adsorb more strongly to mineral surfaces.

Figure 6d explains that the orange colored area corresponds to the total work for the peeling a polymer from the surface [42]. By applying this method to the force curve in Figure 6b (inset), the work per nm peeling of the sample is obtained. By taking the part of the curve between 20 and 80 nm of extension, thus avoiding the complication in the initial part, an average peeling force of 55 pN is obtained (Table 1). Then, the work done to peel 60 nm is equal to 55 × 10^−12^ N times 60 × 10^−9^ m, or 3300 × 10^−21^ J, which is equal to 55 × 10^−21^ J/nm. The total work done to extend the sample from 0 to 80 nm is estimated to be ~4400 × 10^−21^ J. By multiplying these results by Avogadro’s number, 33 × 10^3^ J/nm/mol is obtained. If there are three monomeric residues per nm of the polymer and assuming that all of them participate in the adsorption, the adsorption bond strength is about 10 kJ/mol under the given experimental conditions, which is about 1/30–1/40 of the strength of a typical covalent bond in vacuum, and more typical of a weak non-covalent bond. A summary of the experimental results is given in Table 1.

## 3. Discussion

The interaction of amino acids and mineral surface may have played an important role in chemical evolution leading to the origins of life [12,25]. After the abiotic synthesis of amino acids, there must have been processes that concentrated them sufficiently for polymerization. Certain mineral surfaces might also have catalyzed polymerization. The degree to which concentration or catalysis could occur depends on the strength of amino acid-mineral adsorption. We herein directly measured the strength of these interactions in amino acid-pyrite systems. 

In this study, we first measured the unbinding force of Gly and di-Gly from the surface of pyrite. Although the frequency of observing positive indications of unbinding was low, especially for monomeric Gly, the measured force under the pulling speed of ~5 µm/s was in the range of ~40–60 pN. The magnitude of the unbinding force is in a typical range of separating non-covalently associated molecules, but much smaller than that for covalent bond disruption (~2–3 nN) [43]. The dynamics of bound molecules on solid surfaces are important for the promotion of the subsequent dimerization and polymerization reactions, as described in the introduction. If the unbinding force is too large, bound molecules would not be able to move freely on the solid surface. 

We then turned to tri- and penta-Gly interaction with pyrite surface. The increase of binding frequency for these longer peptides can be partially explained by assuming that the number of binding sites on pyrite is positively correlated with the number of peptide groups in oligomers. As explained above, for *n*-independent binding sites, both on Gly oligomers and on pyrite surface, the statistical factor for the increase in binding frequency will be (2^n^ − 1). Since the AFM tip forcefully pulls and dissociates the interacting pair, dissociation probability is independent of the number of the binding sites. Moreover, if the unbinding of an oligomer takes place sequentially from the unit nearest to the crosslinker to the most distant one, it is not surprising that a large change in the unbinding force between different oligomers was not observed, as in the case of polymer peeling/pull-out for PLL unbinding. Observation of higher force peaks at around 110 and 140 pN in the histogram of penta-Gly (Figure 4b) above the main peak of 70 pN is probably due to the increased rigidity of the molecule. Gly oligomers are known to form helical rod-like structures near to the α-helix site on the Ramachandran plot due to the intramolecular hydrogen bonds [44]. If the molecular rigidity increases, the binding sites may not behave independently from each other, increasing the probability of cooperative unbinding of two or more sites, which should increase the unbinding force significantly. These arguments will apply to the observed case of PLL and PGA over their monomeric counterparts.

**Figure 7:**
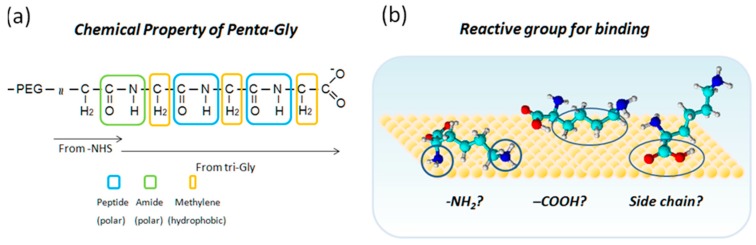
Chemical structure of PEG-tethered penta-Gly. (**a**) Chemical properties of PEG-tethered penta-Gly with an emphasis of length dependent units in boxes in different colors. The amide and peptide groups are similar but the former is formed between amino acids and non-amino acids whereas the latter is between amino acids. Penta-Gly will find polar areas (positively or negatively charged) and/or hydrophobic patches on the pyrite surface for enhanced binding compared with negatively charged monomer Gly, which may occasionally find positively charged sites; and, (**b**) possible factors that may be responsible for the interactions of amino acids with pyrite surfaces.

Figure 7a depicts the chemical structure of peptide, amide and methylene (bridge) groups that constitute the cross-linked part of penta-Gly. In the former two groups, the carbonyl carbon has a partial positive charge, whereas carbonyl oxygen and peptide/amide nitrogen have partial negative charges. Methylene groups are considered hydrophobic. The tri-Gly and penta-Gly parts of the sample have the possibility of interacting with various sites of different electrostatic properties on the pyrite surface. For example, by using a cantilever that is modified with positively charged PLL or negatively charged PGA, the AFM-based force spectroscopy method can probe for local electrostatic nature of the pyrite surface. It is also possible to study the local hydrophobic nature of the pyrite surface if amino acids, such as phenylalanine, are linked to the cantilever. To support this idea, we are collecting binding frequencies and unbinding force of the hydrophobic amino acids, such as phenylalanine (Phe), leucine (Leu), and tyrosine (Tyr) (manuscript in preparation) on a pyrite surface. It is therefore important for future studies to determine correlations between the potential binding sites on peptides and pyrite surfaces.

## 4. Materials and Methods

### 4.1. Natural Pyrite and Amino Acids for AFM Operations

Single crystalline cubes of natural pyrite (mined at Navajun, La Rioja, Spain) were purchased from Crystal World (Kyoto, Japan) and from Planey Co. (Tokyo, Japan) were used in our experiments. This mineral was cut into small slices (1 cm × 1 cm × 2 mm) using a Lab Cutter (MC-120, Maruto, Japan) and a slow Isomet speed saw (Buehler Ltd. 41 Waukegan Road, Lake Bluff, IL, USA). After cutting, the mineral substrates were cleaned, first, by sequential ultra-sonication in acetone, ethanol, and deionized water to remove contaminants from the surface, and then finally activated in 0.01 M HCl for 30 min., followed by washing with Milli-Q water remove any further oxidation. We performed binding studies on both the newly exposed and the original outer surface (will be referred to as “inner” and “outer” surface, respectively, hereafter) of the crystal, both extensively washed, and found no significant differences in the results. The characterization of the pyrite surface was described in our previous report [31]. Gly monomer and its oligomers, lysine (Lys) and poly-l-lysine (PLL), l-Glu, and poly-l-glutamic acid (PGA) were purchased from Sigma-Aldrich (St. Louis, MO, USA) and used without further purification.

Pyrite surfaces are known to oxidize in the presence of O_2_ both under air and in water. Since pyrite’s point of zero charge (PZC) is around pH 2 for acid-cleaned samples and around pH 6 for samples kept in the presence of O_2_, the pyrite surfaces under our experimental conditions (in phosphate buffered saline (PBS) at pH 7.4) must have both positively and negatively charged areas [45]. Figure 1 shows a schematic view of our experimental method. 

### 4.2. Modification of AFM Probes with Gly and Its Oligomers

Two-sided gold-coated triangular silicon nitride cantilevers (part number OMCL-TR400-PB-1), equipped with two triangular cantilevers with nominal force constants *k* of 0.02 and 0.09 nN/nm were purchased from Olympus (Tokyo, Japan). The height of the probe tip of the cantilever was 2.9 µm. To modify the AFM probes with Gly and its oligomers, the gold-coated cantilevers were reacted with a hetero-bifunctional polyethylene glycol (PEG) crosslinker MAL-PEG-NHS with an average MW of 4000 purchased from Sigma-Aldrich, (St. Louis, MO, USA), using published modification methods [46,47,48,49]. 

First, cantilevers were cleaned in a UV ozone cleaner (Filgen NL-UV253, Nippon Laser & Electronics Lab., Tokyo, Japan; or ProCleaner™ Plus system from BioForce Nanosciences Holdings, Inc., Ames, IA, USA). The gold coated AFM cantilevers were reacted with a 10:1 mixture of 6-mercapto-1-hexanol and 1,8-octane-dithiol (both from Sigma-Aldrich, St. Louis, MO, USA), with a total concentration of 1 mg/mL in ethanol for overnight [50]. After washing with ethanol and PBS, the cantilevers were reacted with MAL-PEG-NHS (1 mg/mL in phosphate buffered saline at pH = 7.4), allowing for the alkane dithiol and maleimide groups on the crosslinker to react to form a covalent bond leaving the succinimidyl group on the other end of the crosslinker free for further reaction with amino acid amino groups. 

### 4.3. AFM Imaging & Force-Curve Measurements

A NanoWizard II AFM (from JPK Instruments, Berlin, Germany), equipped with an Axio-observer D1 inverted microscope (Carl Zeiss, Jena, Germany) and an MFP-3D^TM^ Stand Alone AFM (Asylum Research, an Oxford Instruments Company, Santa Barbara, CA, USA) were used for the AFM imaging and force measurements. The AFM head has a 15-µm *Z*-range linear piezoelectric scanner and an infrared laser in the optical lever system for the detection of cantilever deflection. The sensitivity of the optical lever system was calibrated and the cantilever spring constant was determined in situ before or after every experiment, using the built-in software of the AFM system based on the thermal noise method [51,52]. Within the reported uncertainty of this method (10%), cantilever spring constants were found to agree with the manufacturer’s specifications.

A closed loop system was used to control the speed and positioning of the vertical piezo elements [53]. All of the imaging and force spectroscopy experiments were performed at room temperature (25 °C) under PBS at pH 7.4. The cantilever position and its vertical deflection were recorded in the AFM force mode.

In recent years, a fast scanning AFM has been applied for force measurement experiments [54,55,56,57]. We expect that the force mode of the AFM will be widely applied in biological investigations including those involving mineral surface vs. biological molecule interactions.

## Figures and Tables

**Table 1:** MPF* and relative frequency of interaction.

Molecular Species	MPF * Obtained by AFM (pN)	Relative Frequency of Interaction (%)
Gly	− **	<1
Di-Gly	50	3‒5
Tri-Gly	55	7‒10
Penta-Gly	76, 110, 140	10‒15
Lys	57	10‒15
PLL	55	15‒20
Glu	− **	<1
PGA	65	20‒25

* MPF: Most Probable Force obtained as the mode of Gaussian fitting curves to individual histograms. The error range of MPF was estimated to be within ±5%. **: interactions were only rarely observed.

## Abbreviations

Gly	glycine
di-Gly	diglycine
tri-Gly	triglycine
penta-Gly	pentaglycine
Lys	lysine
pLL	poly- l-lysine
Glu	glutamic acid
PGA	polyglutamic acid
PBS	phosphate buffered saline
MPF	most probable force
MAL-PEG-NHS	Poly(ethylene glycol) ( *N*-hydroxysuccinimide 5-pentanoate) ether *N*′-(3-maleimidopropionyl)aminoethane
